# Supplementary figures and images for: Nitrous oxide variability at sub-kilometre resolution in the Atlantic sector of the Southern Ocean
Source: PeerJ. 2018 Jul 6;6:e5100. doi: 10.7717/peerj.5100 (PMC6037155; doi:10.7717/peerj.5100)

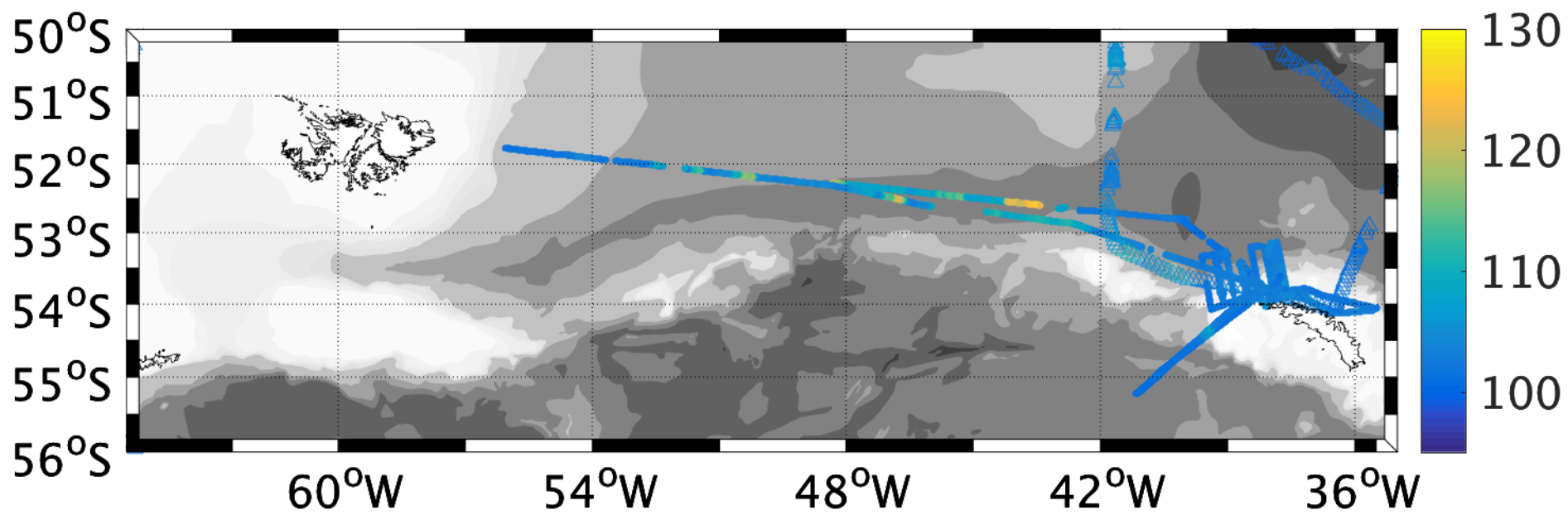

Supplement: Supplemental Information 1 — All N2O saturation data for the survey area 50 to 56°S and 35 to 65°W. Data from JR260B displayed as filled circles, previously published data (Weiss et al., 1992) as open triangles. [file peerj-06-5100-s002.pdf]

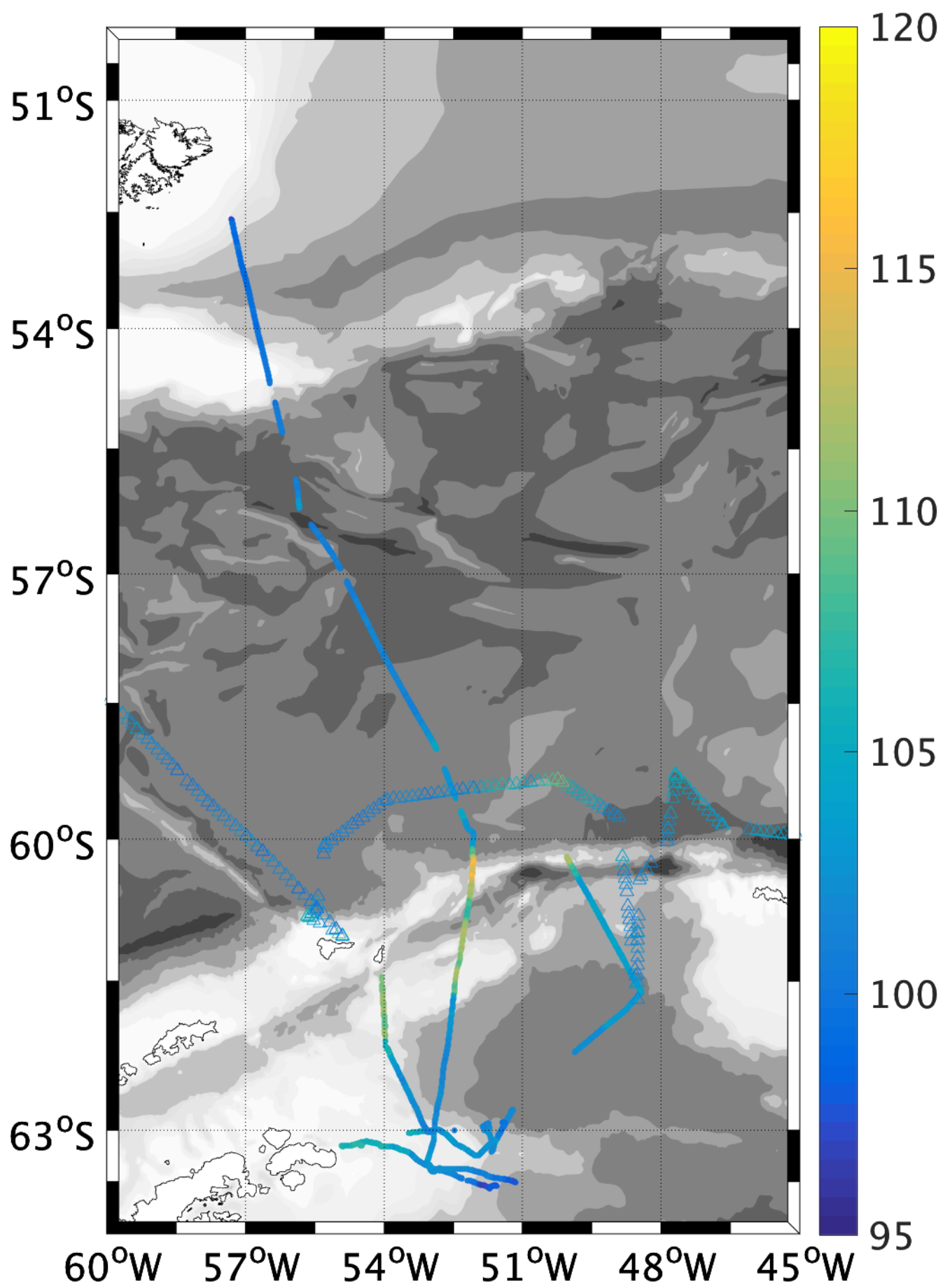

Supplement: Supplemental Information 2 — All N2O saturation data for the survey area 50 to 64°S and 45 to 60°W. Data from JR255A/GENTOO displayed as filled circles, previously published data (Weiss et al., 1992) as open triangles. [file peerj-06-5100-s003.pdf]
